# Several Characteristics of *Oidiodendron maius* G.L. Barron Important for Heather Plants’ Controlled Mycorrhization

**DOI:** 10.3390/jof9070728

**Published:** 2023-07-05

**Authors:** Vyacheslav S. Mikheev, Irina V. Struchkova, Ludmila M. Churkina, Anna A. Brilkina, Ekaterina V. Berezina

**Affiliations:** 1 Department of Biochemistry and Biotechnology, Institute of Biology and Biomedicine, Lobachevsky State University of Nizhny Novgorod, Gagarin Avenue 23, 603950 Nizhny Novgorod, Russia; 2 LLC “Mikofit”, Internatsionalnaya 56b, 606440 Bor, Russia

**Keywords:** ericoid mycorrhiza, colony growth, flavonoids, protease, phenol oxidase, phytase, auxin

## Abstract

*Oidiodendron maius* G.L. Barron is a recognized fungal species capable of forming ericoid mycorrhiza with various positive effects on host plants; therefore, newly found and previously uncharacterized *O. maius* strains may be valuable for heather plants’ controlled mycorrhization. Characteristics of the *O. maius* F3860 strain were studied, i.e., mycelium growth on various nutrient media and the ability to secrete auxins and enzymes. *O. maius* F3860 grew rapidly on malt extract agar and potato dextrose agar. It was also able to grow on nutrient media suitable for heather plant cultivation. The presence of the flavonoids rutin and quercetin increased the mycelium growth rate compared to the control, starting from the 8th to the 13th days of cultivation. The ability to secrete auxins was confirmed with bioassay and thin-layer chromatography, and their content, as well as phytase activity, was estimated spectrophotometrically. Both in nutrient media with tryptophan and without it, *O. maius* F3860 secreted about 6 μg IAA/mL growth medium. *O. maius* F3860 possessed extracellular phytase, protease, and phenol oxidase activities. The investigation indicates *O. maius* F3860’s promise for heather seedling inoculation as an approach to increase their fitness.

## 1. Introduction

Fungi that form a symbiosis with Ericaceae family representatives improve the host plants’ mineral nutrition and protect them from various stresses. Thus, these fungi may contribute to maintaining natural ecosystem stability and increasing agricultural product yield [1,2,3,4,5]. Anamorphic ascomycete *Oidiodendron maius* G.L. Barron (1962) (Myxotrichaceae, Leotiomycetes) is one of the main fungi of ericoid mycorrhiza. Its mycorrhiza-associated development in plant roots has been repeatedly confirmed with culture-dependent and/or culture-independent methods in *Vaccinium myrtillus* L. [6,7], *V. uliginosum* L., *V. corymbosum* L. [8,9], and other heather species [3,10,11]. As a model organism, *O. maius* is used to study mycorrhiza evolution, organization, and functioning, as well as mechanisms of heavy metals tolerance [6,7,12,13]. *O. maius* mutants are used to directly observe spatial details of the mycorrhization process [14] and clarify the role of individual enzymes in fungal metabolism [15,16].

Along with a symbiotic lifestyle, *O. maius* is capable of saprotrophic and endophytic existence in the roots of non-host plants, for example, *Quercus ilex* and *Arabidopsis thaliana* [1,13,17,18]. Genome sequencing of *O. maius* leads to a clearer understanding of saprotrophic and mutualistic traits interweaving in this fungus [13].

A significant number of issues related to *O. maius* and its interaction with host plants and the environment in general remain unexplored. Moreover, *O. maius* strains can differ significantly in their value as an inoculant, so not all of them can be equally used in heather plant biotechnology. Preliminary confirmation of the potential of the fungus proposed for inoculation is relevant due to the longevity and high labor intensity of inoculated heather plant cultivation. Thus, mycelium growth on various nutrient media is necessary to obtain standardized fungal material in the form of plugs cut from the marginal zone of the colony, as this material is convenient for studying features of symbiotic relationship formation between plants and fungi under model conditions in vitro and ex vitro. In addition, it is important to know the peculiarities of fungal growth on the medium on which host plants are cultivated and in the presence of phenolic compounds abundant in heather plant tissues. Until now, *O. maius*’ response to flavonoids that possess both inhibitory and stimulatory effects on other fungal species has not yet been studied. Moreover, it is necessary to know the composition of the fungal-secreted enzymes and hormones that may take part in penetrating into plant tissues and strengthening the fungal–plant partnership due to host plant nutrition and growth improvement. Since newly found *O. maius* strains may be valuable in plant biotechnology for increasing heather plants’ fitness, the aim of this investigation was to study the biochemical and physiological characteristics of the *O. maius* F3860 strain, which are important for plants’ controlled mycorrhization. These characteristics include mycelium growth on various nutrient media and the ability to secrete enzymes and auxins.

## 2. Materials and Methods

### 2.1. Oidiodendron maius F3860 Strain as an Object of Investigation

*Oidiodendron maius* G.L. Barron VKM F3860 strain (hereinafter referred to as *O. maius* F3860) was obtained from the All-Russian Collection of Microorganisms (G.K. Skryabin Institute of Biochemistry and Physiology of Microorganisms, Russia). It was found by A.V. Aleksandrova (Moscow State University, Russia) on the East European Plain from the A1 horizon of podzolic soil in a pine–spruce forest on the banks of the Volga River. The species identity was confirmed by identification using sequencing of diagnostic loci and comparing the established sequences of the DNA-ITS region with the reference sequences in GenBank (NCBI) using BLAST, carried out in the laboratory of the All-Russian Research Institute of Agricultural Biotechnology (Supplementary Dataset S1). *O. maius* F3860 showed the maximum (100%) level of similarity of ITS sequences with the *O. maius* strain isolated in Sweden (ID: MH860824.1), over 99% similarity with strains isolated in Canada (ID: MT028061.1) and the USA (ID: MH864347.1) [19,20], and less than 97% similarity with the isolate found in the Samara region (Russia) in *Orthilia secunda* roots [21]. *O. maius* F3860 was maintained on potato dextrose agar (PDA).

### 2.2. Influence of Nutrient Media Composition on O. maius F3860 Colonies Growth

To determine the rate of enlargement of colony diameter and to identify other cultural features, *O. maius* F3860 was grown on agar nutrient media of various compositions: Czapek-Dox agar (CDA), malt extract agar (MEA), and potato dextrose agar (PDA), which are widely used in mycology [22,23], as well as Anderson medium (And) and Woody Plant medium (WPM), which are most commonly used for heather plant in vitro cultivation [4,24]. The pH values of CDA, MEA, and PDA nutrient media were 5.5, and those of WPM and And were 5.0.

To study the effect of plant metabolites on *O. maius* F3860 growth, modified CDA media were used, in which either the flavonoids quercetin or rutin (quercetin 3-O-rutinoside) (Acros Organics, Belgium) in the form of 50% ethanol solutions to a final concentration of 10 μM (CDA + Q and CDA + R media, respectively), or the same volume of 50% ethanol (control medium CDA + EtOH) were introduced.

Inoculation with fungal spores was performed by injection in the center of a Petri dish (D = 9 cm) containing 13 ± 2 mL of nutrient medium. Cultivation was carried out in the dark at 22 ± 2 °C for 22 days, with periodic measurements of colony diameter. Colony diameter was measured in two perpendicular directions, and the average of these measurements was obtained. In two independent experiments, 5 colonies grown on each type of medium were analyzed. The rates of enlargement of colony diameter (mm/day; K_D_) on different media were calculated as linear regression slopes after linear approximation of average diameters for each cultivation period, starting from the fourth day after inoculation. The reliability parameter for linear approximation R^2^ in all cases was above 0.92.

### 2.3. Identification of O. maius F3860 Extracellular Enzyme Activities

The ability of *O. maius* F3860 to produce other extracellular enzymes was studied with semiquantitative plate methods. Inoculation with fungal spores was performed by injection in the center of a Petri dish containing a nutrient medium with a substrate for each enzyme. Milk agar (60 mL of skim milk and 180 mL of 3% aqueous agar) enlightenment reaction was used to detect proteolytic activity [23]. To detect lipolytic activity, Tween-80 was used as a water-soluble analog of natural lipids (10 g of peptone, 5 g of NaCl, 0.1 g of CaCl_2_ × 2 H_2_O, 20 g of agar per 1 l of the medium; an autoclaved solution of Tween-80 was added to the autoclaved medium at a rate of 10 mL per 1 L). Lipolytic activity is determined by nutrient medium transparency loss in the zone of lipase action [23]. Phenol oxidase activity was determined with a modified Bavendamm reaction using tannic acid or tetramethylbenzidine (TMB) as indicator compounds (5 g of tannic acid or 1 g of TMB per 1 l of PDA medium). Phenol oxidase activity is determined by the appearance of reddish-brown (in the case of tannic acid) or blue (in the case of TMB) zones in the medium around the fungal colony [25].

The appearance of color or enlightenment zones in media was assessed on the seventh day of cultivation. The experiments were carried out in triplicate.

The ability of *O. maius* F3860 to hydrolyze organic phosphorus compounds was assessed by phytase activity in culture filtrate. *O. maius* F3860 was cultivated on CDB under stationary conditions in the dark at 22 ± 2 °C for three weeks. Inoculation was carried out by introducing one mycelium plug (d = 8 mm) into a 100 mL flask containing 70 mL of culture broth.

Phytase activity was determined in culture filtrate on the 14th and 21st days of cultivation, using Na-phytate, as previously described [4]. The determination was carried out in three biological and eight analytical replicates.

### 2.4. Bioassay for Identification of O. maius F3860 Ability to Synthesize Auxins

To assess the auxin-like action of *O. maius* F3860 exometabolites, a bioassay was performed according to [26] using fungal culture broth and a model object common bean *Phaseolus vulgaris* L. cv. ‘Red Kidney’ cuttings (Southern Rice Company, Russia).

*O. maius* F3860 was cultivated under stationary conditions in the dark at 22 ± 2 °C on Czapek-Dox broth (CDB) and its modification, additionally containing L-tryptophan (JRPC, Shijiazhuang, China) at a final concentration of 2.5 mM (CDBtrp medium). Then, 5 mL of spore suspension (10^6^/mL) was added to a 500 mL flask containing 250 mL of medium. After 14 days, mycelium was filtered and dried, and the absolute dry weight was determined. The obtained filtrates of culture broths without tryptophan (CF) and with tryptophan (CFtrp) were used for bioassay.

Common bean seeds were surface sterilized in 15% H_2_O_2_ solution for 30 s and pre-germinated in sterile distilled water under natural light conditions at 22 ± 2 °C. Cuttings 12–14 cm long with a pair of true leaves were cut from 10-day-old seedlings and kept for 6 h in one of the following solutions: (a) water (H_2_O variant); (b) Czapek-Dox broth, in which fungus was not inoculated (CDB-0); (c) CF; (d) CFtrp. There were 10 cuttings per solution. After incubation in solutions, cuttings were rinsed with sterile water and rooted in water under natural light conditions at 22 ± 2 °C. The water was changed daily. For each cutting, the time of the root appearance was recorded. Additionally, the root number and length of stem segments on which roots appeared were recorded 11 days after the first root formation. The experiment was carried out twice.

### 2.5. Determination of Indole Acetic Acid or Analogues with Salkowski Reagent

Indole compounds (indole acetic acid (IAA) and analogs) in CF and CFtrp were determined chromatographically and spectrophotometrically using Salkowski reagent.

For chromatographic determination, filtrates CF and CFtrp were acidified to pH 3.0, combined with ethyl acetate at a ratio of 1:2, stirred for 10 min, and kept at −20 °C overnight, then filtered in cold conditions to remove ice crystals and evaporated on an EYELA N-1000 rotary evaporator (EYELA, Tokyo, Japan) [27]. The almost dry residues of CF and CFtrp were dissolved in 3 mL of methanol and designated as CF extract and CFtrp extract, respectively.

Separate tracks of ALUGRAM^®^ Xtra SIL G/UV254 TLC plates (Macherey-Nagel, Düren, Germany) were loaded with 25 μL of CF and CFtrp extracts, as well as a mixture of IAA (Sigma, Shanghai, China) and L-tryptophan (600 μg/mL each). Chromatography was carried out in the solvent system *n*-hexane:ethyl acetate:isopropanol:acetic acid = 40:20:5:1 [28]. Salkowski reagent was used for indoles visualization.

The content of indole compounds in CF and CFtrp extracts was determined spectrophotometrically using Salkowski reagent [29]. Colored probes’ optical density was measured on an Epoch microplate spectrophotometer (Biotek, Shoreline, WA, USA) at λ = 530 nm. The content of total indole compounds per 1 mL of culture liquid and per 1 g of mycelium dry weight was calculated using IAA as a standard (concentration of IAA stock solution was 70 μg/mL). The determination was carried out in three biological and three analytical replicates.

### 2.6. Statistics

Statistical analysis was conducted using Microsoft Excel 2010. One-way analysis of variation (ANOVA) was used to evaluate differences in diameters of colonies grown on different nutrient media, in root number and length of stem segments on which roots appeared in a bioassay with common bean, in IAA concentration, and in phytase activity. Significant differences were evaluated at *p* < 0.05.

## 3. Results

### 3.1. Influence of Nutrient Media Composition on O. maius F3860 Colonies’ Growth

**Figure 1:**
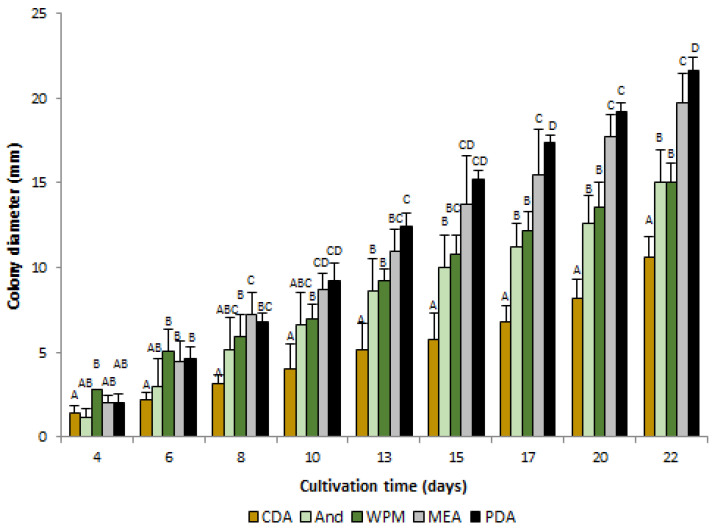
*O. maius* F3860 colonies diameters on different nutrient media. CDA—Czapek-Dox agar, And—Anderson medium, WPM—Woody Plant medium, MEA—malt extract agar, PDA—potato dextrose agar. Different letters indicate statistically significant differences in the diameters of colonies grown on different media, *p* < 0.05.

Among the tested nutrient media widely used in mycological studies and heather plant biotechnology, the maximum diameter of *O. maius* F3860 colonies at the end of the cultivation period (on the 22nd day of growth) was observed on PDA medium (Figure 1); the rate of enlargement of colony diameter (K_D_) was 1.0878 (Table 1). Diameters of colonies of the same cultivation time on other media were smaller and amounted to the values on PDA: for MEA, 91%; for WPM and And, 69%; and for CDA, 51%. From the 13th day of cultivation, the diameters of colonies on the CDA medium were significantly smaller than on all other media; the K_D_ value was 0.4661. The diameters of colonies on WPM and And media did not differ during the cultivation period, and the growth of colonies on these media was characterized by close K_D_ values, 0.6612 and 0.7230, respectively.

Sporulation on CDA, WPM, and PDA media occurred on the 15th day of cultivation and on And medium on the 10th day of cultivation. Sporulation on the MEA medium was not observed during the experiment.

**Figure 2:**
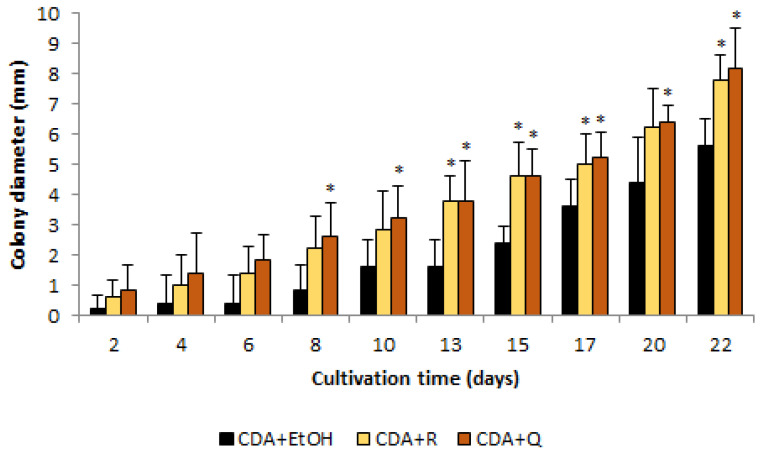
*O. maius* F3860 colonies diameters on modified Czapek-Dox agar media. CDA + EtOH—Czapek-Dox agar with ethanol, CDA + R—Czapek-Dox agar with rutin ethanolic solution (final concentration of 10 μM), CDA + Q—Czapek-Dox agar with quercetin ethanolic solution (final concentration of 10 μM). *—colony diameter on medium with flavonoid is statistically bigger than those on CDA + EtOH, *p* < 0.05.

In the presence of flavonoids, the diameters of colonies, starting from the 8th (for quercetin) and the 13th (for rutin) days of cultivation, were about 1.7 times greater than in control (CDA + EtOH; Figure 2). The maximum difference was recorded on the eighth day of cultivation when the diameter of colonies on CDA + Q medium exceeded control by 225%.

### 3.2. Identification of O. maius F3860 Extracellular Enzyme Activities

**Figure 3:**
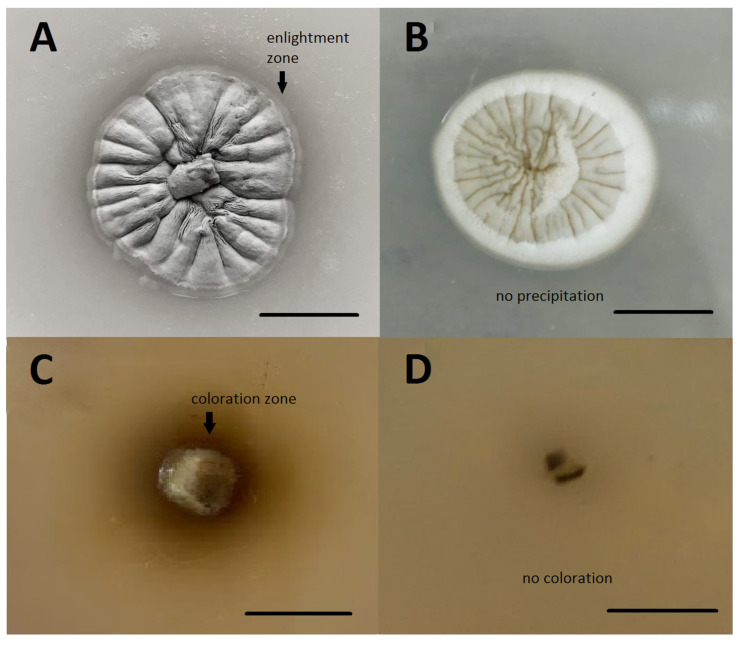
Appearance of color or enlightenment zones in media around *O. maius* F3860 colonies due to extracellular enzymatic activity: (**A**) proteolytic activity; (**B**) lipolytic activity; (**C**) phenol oxidase activity (tannic acid as a substrate); (**D**) phenol oxidase activity (tetramethylbenzidine as a substrate). Bar = 1 cm.

The studied strain demonstrated proteolytic activity. During cultivation on milk agar, enlightenment zones formed around fungal colonies, which is associated with the hydrolysis of milk casein (Figure 3).

*O. maius* F3860 did not possess lipolytic activity, and its growth on medium with Tween-80 did not lead to precipitation of calcium salts of fatty acids around fungal colonies.

During cultivation on medium with tannic acid, zones were formed around *O. maius* F3860 colonies, colored reddish-brown with products of tannic acid enzymatic oxidation. This indicates the ability of *O. maius* F3860 to produce laccase and/or tyrosinase. However, the absence of color change in medium with TMB indicates the absence of laccase activity.

*O. maius* F3860 also demonstrated phytase activity, which was 7.4 ± 1.5 U on the 14th day of cultivation. On the 21st day of cultivation, it significantly decreased and amounted to 5.2 ± 1.5 U.

### 3.3. Bioassay for Identification of O. maius F3860 Ability to Synthesize Auxins

**Figure 4:**
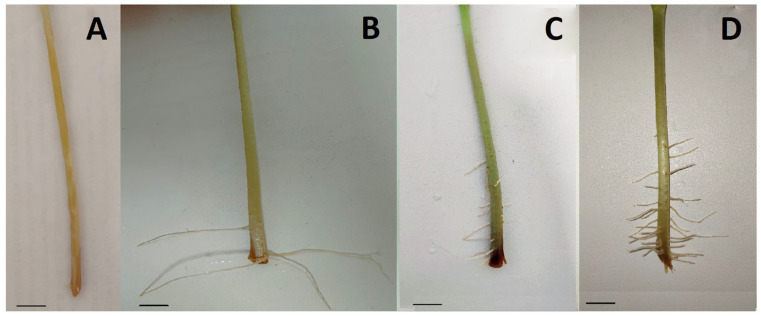
Common bean cuttings treated with different solutions 11 days after first root formation: (**A**) treatment with water (H_2_O); (**B**) treatment with Czapek-Dox broth, in which fungus was not inoculated (CDB-0); (**C**) treatment with culture filtrate of Czapek-Dox broth (CF); (**D**) treatment with culture filtrate of Czapek-Dox broth with tryptophan (CFtrp). Bar = 1 cm.

**Figure 5:**
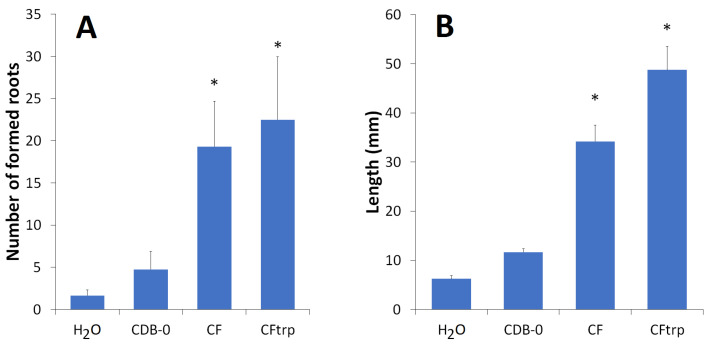
Influence of *O. maius* F3860 culture filtrates on common bean cuttings root formation: (**A**) number of formed roots; (**B**) length of stem segments on which roots appeared. *—number of formed roots and length of stem segments on which roots appeared in CF and CFtrp variants are statistically bigger than those in H_2_O and CDB-0 treatments, *p* < 0.05.

The use of *O. maius* F3860 culture filtrates accelerated the appearance of roots in common bean cuttings. Thus, after cuttings’ treatment with CF or CFtrp, they appeared on the 5th day, while after treatment with CDB-0 and H_2_O, they appeared on the 7th and 11th days, respectively (Figure 4). In addition, both CF or CFtrp treatments increased the number of formed roots more than three times compared to CDB-0 treatment and more than 6 times compared to H_2_O treatment (Figure 5A), and the length of stem segment on which roots appeared, as well (Figure 5B).

### 3.4. Determination of Indole Acetic Acid or Analogues with Salkowski Reagent

Both in the presence of tryptophan and without it, *O. maius* F3860 secreted indole compounds into culture broth, the total concentration of which was 6.03 ± 0.96 μg/mL of CFtrp and 6.42 ± 0.58 μg/mL of CF (respectively, 2.71 ± 0.05 μg/g dry mycelium weight and 2.60 ± 0.04 μg/g dry mycelium weight).

**Figure 6:**
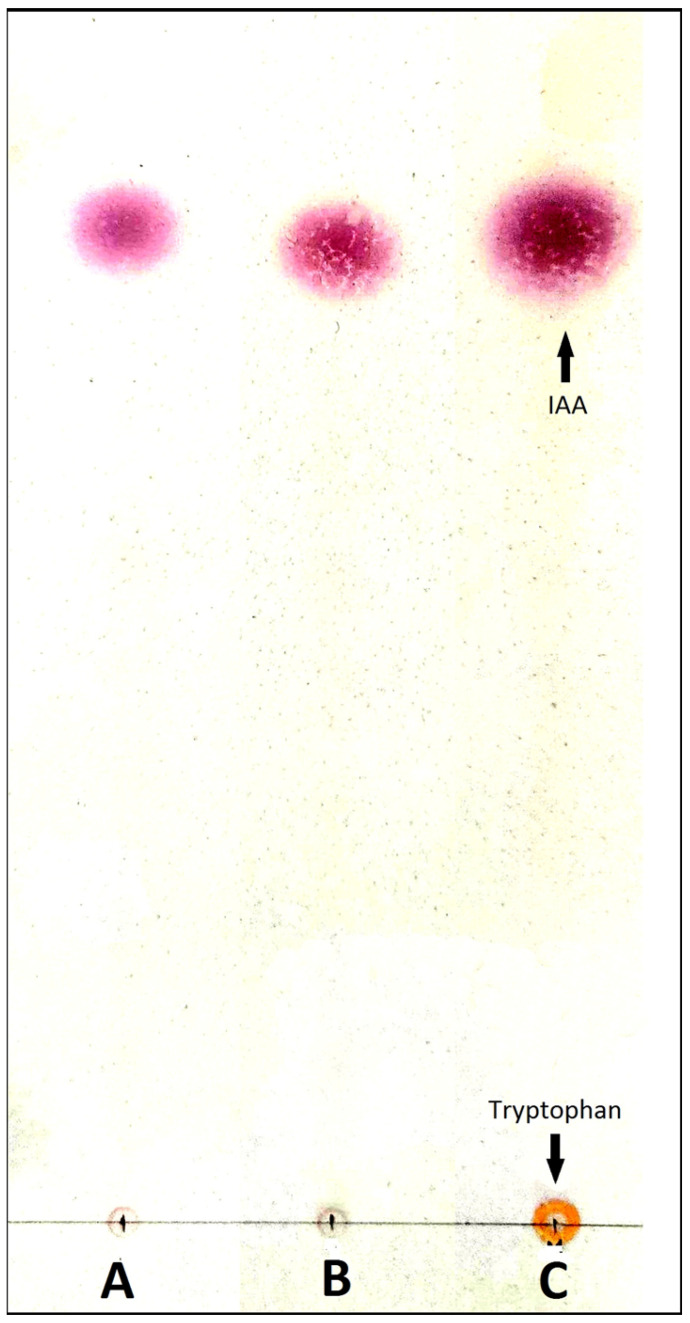
Sample chromatogram of *O. maius* F3860 indole compounds in ethyl acetate extracts of culture filtrate of Czapek-Dox broth with tryptophan (CFtrp extract; track A) and of culture filtrate of Czapek-Dox broth (CF extract; track B); track C—indole acetic acid (IAA) and L-tryptophan standards (600 μg/mL each). Thin-layer chromatography, ALUGRAM^®^ Xtra SIL G/UV254 (Macherey-Nagel, Germany) plate, solvent system *n*-hexane:ethyl acetate:isopropanol:acetic acid = 40:20:5:1, visualization with Salkowski reagent.

Thin layer chromatography of CF and CFtrp extracts revealed indoles’ presence, the Rf value (0.74) and the color of which corresponded to IAA (Figure 6).

## 4. Discussion

### 4.1. Influence of Nutrient Media Composition on O. maius F3860 Colonies Growth

In plant biotechnology, when using mycorrhizal fungi for plant inoculation in both in vitro and ex vitro conditions, it is important to study each specific strain in advance since different strains of the same fungal species can differ significantly in cultural characteristics, including the rate of enlargement of colony diameter on medium, even if its formulation is unchanged [6,30]. Among the tested media, *O. maius* F3860 rapidly grew on PDA and MEA nutrient media. It was also able to grow on And and WPM nutrient media which are used for heather plant in vitro cultivation. The maximum colonies’ diameter achieved after 22 days of cultivation did not exceed 22 mm (observed on PDA). This indicates the *O. maius* F3860 strain’s slow development and is comparable with the diameter of colonies of several *O. maius* strains from Poland, which varied from 22 to 36 mm after 24 days of cultivation [6] and with the growth rate of *O. maius* strains from Taiwan (0.74–1.24 mm/day) [30]. The *O. maius* strain from China had a somewhat higher growth rate; its colonies’ diameter varied from 20 to 25 mm after 14 days of cultivation on MEA [3]. Therefore, *O. maius* is a slow-growing fungus compared to, for example, fungi from phylum Mucoromycota, for which the growth rate on PDA may exceed 6 mm/day [31].

For successful colonization of tissues of the host plant and further co-existence with it, *O. maius* has to successfully resist host defense compounds, in particular, flavonoids. Flavonoids represent a large group of secondary metabolites of phenolic origin that possess various functions within plants and in interactions between organisms [32,33]. They are abundantly synthesized by heather plants [34,35], remaining in tissues and released into the soil (or nutrient medium) in the form of exudates, leaf litter, dead roots, etc., and, therefore, affect *O. maius* both during its saprotrophic and symbiotic lifestyle.

Antifungal effects of flavonoid quercetin have previously been noted in *Aspergillus* spp. (at a concentration of 0.4 mM) [36,37] and *Candida* spp. (2 mM and higher) [38]. Rutin (quercetin 3-O-rutinoside) is also known to possess antifungal effects against *Candida krusei* [39].

As for some phytopathogenic fungi, it was shown that plant flavonoids did not have an inhibitory effect on them. For example, quercetin in the concentration of 50 mM did not decrease the growth of different *Colletotrichum* isolates [40]. At the same time, quercetin and rutin have been shown to be able to accelerate plant symbionts’ growth and development. For example, germination of spores of ectomycorrhizal fungus *Tuber borchii* was stimulated by quercetin concentrations up to 4 μM [41]; spore germination, mycelium growth, and roots colonization of several species of arbuscular mycorrhizal fungi were stimulated by quercetin concentrations from 5 µM [42] to 8 µM of *Gigaspora margarita* [43].

In our studies, the flavonoids rutin and quercetin had no inhibitory effect on *O. maius* F3860; on the contrary, in the presence of these flavonoids (10 μM), fungal growth was accelerated. The absence of inhibition may be connected with fungal enzymes that cleave the carbon skeleton of flavonoid aglycone or otherwise inactivate it. For *Aspergillus* spp., *Penicillium* spp., and some other species, the ability to use rutin as the sole source of carbon and energy in the rutin catabolic pathway, which involves glycosidases, dioxygenases (quercetinases), and esterases [44] has been described. Thus, the observed growth acceleration of *O. maius* F3860 colonies may be associated with the use of flavonoids as an additional source of nutrition; however, there are currently no data on the functioning of the rutin catabolic pathway in *O. maius*.

Undoubtedly, faster colony growth does not necessarily mean faster biomass accumulation; it is possible to accelerate growth by reducing hyphae branching and, consequently, the formation of a thinner mycelium mat. However, a rapidly growing colony, although having a slightly branched mycelium, may reach the host plant faster (especially in in vitro conditions), which, in turn, will accelerate plant root colonization.

### 4.2. Identification of O. maius F3860 Extracellular Enzyme Activities

In addition to cultural characteristics, fungi may have different enzymatic potential. In particular, the level of protease production may help in the differentiation of fungi as pathogens and saprotrophs. Saprotrophs do not actively produce proteases for their needs in nitrogen, while pathogens actively produce proteases to penetrate plant tissues [45]. The ability of several *O. maius* strains to hydrolyze proteins was previously shown [13]. The plate test allowed us to establish the presence of proteolytic activity in *O. maius* F3860; however, the size of enlightenment zones visible on milk agar, which was narrow, confirmed low protease activity. The revealed circumstance supports the point of view that *O. maius* does not show typical signs of a phytopathogen [45].

Lipases, like proteases, may be necessary for fungi to degrade plant cell membrane components when fungi penetrate plant tissues. Thus, it is described that *O. maius* lipolytic activity (carboxyesterase B), which, however, appears only after interaction with the host plant [13]. In the absence of the inducer, we did not detect lipolytic activity in *O. maius* F3860.

The ability of fungi to enzymatically oxidize phenolic compounds under natural conditions may be associated with the need for carbon and energy (for example, in lignin-degrading fungi), with the synthesis of melanin, a dark-colored pigment of the fungal cell wall, as well as with detoxification of phenolic compounds, which are protective substances and abundantly presented in plants [46]. For heathers, phenolic compounds are the main compounds that provide plants with protection against pathogenic microorganisms; therefore, fungi interacting with heather plants must have enzymatic counter-protection mechanisms. The ability to metabolize tannic acid, a phenolic polymer, is characteristic of many ericoid mycorrhizal fungi, such as *Meliniomyces* sp., *Leohumicola* sp., *Acremonium implicatum*, and *Cryptosporiopsis ericae* [47]. For some *O. maius* strains, it has also shown the ability to oxidize tannic acid [48], and for other strains, laccase-encoding genes were found [49]. For laccase, the induction of synthesis in the presence of lignin and tannins in medium has been described [50]; however, in our experiment, in the presence of tannic acid or TMB in medium, no induction of laccase synthesis was found, which indicates the ability of *O. maius* F3860 to secrete phenol oxidase but not laccase.

The ability to mobilize inorganic phosphorus from soil compounds in the form of phosphate ions is an important characteristic of fungi used for heather plants’ mycorrhization. This ability associated with organic acids has been repeatedly described for *O. maius* [51,52]; however, in most heather plant habitats, soils contain phosphorus mainly in the form of organic compounds. It is known that among organic phosphorus sources, up to 80% can be accounted for by phytates, which are salts of phytic acid [53]; therefore, to obtain phosphorus, fungi need phytases. Phytases are a group of phosphatases distinguished by their specificity for phytates as preferred substrates and their capability of cleaving phosphates from them.

*O. maius* F3860 produced phytase under our cultivation conditions; thus, it is able to release phosphorus from phytates and, as a result, facilitate the availability of phosphorus to plants. Phytase was active at pH 4.5, which corresponds to the pH of acid soils from natural habitats of heather plants and ericoid mycorrhizal fungi [54,55]. Previously, phosphatase and phytase activity was also found in other root fungi of heather plants, for example, in *H. ericae*, which was able to grow on phytate [56], in dark septate endophytes *Phialocephala fortinii*, *P. glacialis*, *P. turiciensis*, and *Acephala applanata* [4,57]. At the same time, we carried out a quantitative assessment of *O. maius* phytase activity for the first time. The value of specific enzyme activity in *O. maius* F3860 culture broth is comparable to that of *P. fortinii* DSE2, which we determined earlier using a similar method (7.4 U on the 14th day of cultivation and 6.9 U on the 21st day of cultivation, respectively) [4].

The activity of proteases, lipases, phenol oxidases, and phytases has several implications for mycorrhiza-forming *O. maius* fungi. On the one hand, these enzymes hydrolyze soil components and oxidize hardly decomposable compounds in soil complexes. Soil complexes have an irregular structure and include different compounds, which in many cases are strongly interconnected and may interfere with an attack of specialized enzymes on other compounds. Polyenzymatic action leads to the simplification of the structure of the attacked compounds, their complete mineralization, or facilitating the attack on them with the next set of enzymes. On the other hand, these enzymes may be necessary when penetrating into plant tissues for the degradation of cell membrane proteins and lipids. A variety of extracellular enzymes can strengthen the fungal–plant partnership, especially in ecosystems where nutrients are mostly available in organic forms, and also allow ericoid mycorrhizal fungi to function both as symbiotrophs and as saprotrophs [1,2]. Since these fungi are of relatively recent evolutionary origin [13], they may be at a transitional stage from a predominantly saprotrophic lifestyle, which may be related to the transitional role of secreted enzymes [2,55].

### 4.3. Assessment of the O. maius F3860 Strain’s Ability to Synthesize IAA or Analogues

The ability to synthesize auxins is a valuable characteristic of fungi used for controlled mycorrhization. IAA is a key signal that regulates plant root system development and changes its architecture due to lateral root formation on the main root, adventitious root formation on the stem, and root hair formation from the epidermis and their elongation [58]. It is known that auxins also participate in symbiotic relationship establishment between plants and fungi. In particular, it was shown that arbuscular mycorrhizal fungi *Funneliformis mosseae* producing auxin increased root system development of *Poncirus trifoliata* [59]. Thus, auxin-induced development of lateral roots and root hairs leads to an increase in root surface area and is important for enhancing plant mineral and water nutrition and for increasing root colonization by symbionts. At the same time, IAA effects depend on this auxin concentration: primary root elongation is stimulated by low concentrations and inhibited (through stimulation of ethylene formation) by higher concentrations [60].

The positive effects of *O. maius* F3860 CF and CFtrp treatment were shown in our experiment on common bean cuttings’ rooting. These include a shortening of the time of root appearance, an increase in root number, and in the length of stem segments on which roots appeared. This clearly confirms the ability of the strain to synthesize exometabolites of an auxin nature.

In fungi, IAA synthesis is possible in two ways: tryptophan-dependent and tryptophan-independent [58,61]. In the case of the tryptophan-dependent pathway, tryptophan functions as a precursor in IAA biosynthesis. It is assumed that in *O. maius,* the tryptophan-dependent pathway functions [62], but complete clarity on this issue has not yet been achieved. In connection with the given information, to identify the ability to synthesize IAA, we used culture broth with and without L-tryptophan, and IAA synthesized by *O. maius* F3860 was detected on both medium types. This fact may be related to high tryptophan concentration in *O. maius* mycelium [62]. It is this stock of precursor that is apparently used for IAA synthesis on culture broth without tryptophan. At the same time, the fungus was able to absorb tryptophan from CDBtrp medium, as evidenced by the absence of a spot similar to tryptophan in the chromatogram (Figure 4), but this ability did not increase IAA excretion.

The ability to synthesize IAA was found in the endophytes of various plants. Thus, it was found in Solanaceae and Phyllanthaceae families endophyte *Fusarium oxysporum* (6–24 µg/mL) [63,64], coffee tree endophyte *Colletotrichum fructicola* (about 800 µg/mL) [65]. There is evidence that *O. maius* produces IAA in amounts ranging from 0.1 µg/g dry mycelium weight on the 7th day of cultivation [62] to 255 µg/g dry mycelium weight on the 12th day of cultivation [18]. Our data supplement the mentioned data, characterizing IAA content in *O. maius* F3860 culture filtrate. In our opinion, it is the amount of IAA released into culture broth that gives the best idea of its beneficial effect on plants. IAA concentration found in *O. maius* F3860 culture filtrate (about 6 µg/mL) is of the same order as such used for ericoid plants cuttings (5–10 µg/mL) [66].

## 5. Conclusions

Our studies of the *O. maius* F3860 strain indicate the prospects for its use in the biotechnology of heather plants. This conclusion is based on the confirmation of a number of features of this strain that are important for successful interaction with host plants. First, the ability of *O. maius* F3860 to grow on media used for heather plant cultivation (And and WPM). Secondly, *O. maius* F3860 is able to grow (and even accelerate mycelium growth) in the presence of flavonoids with protective functions, such as rutin and quercetin. To our knowledge, there is no information about flavonoids’ effect on *O. maius* growth. Growth acceleration may be associated with the signaling function of these compounds in the process of plant–fungus interaction or with their use as an additional source of nutrition, but this aspect requires further study. Additionally, the ability of *O. maius* F3860 to oxidize tannic acid indicates that this strain has enzymatic mechanisms for polymeric phenolic compound degradation. Such mechanisms are possibly aimed at detoxification or involving phenolic compounds in fungal metabolism as a source of nutrition. Thirdly, *O. maius* F3860 is able to secrete in small quantities enzymes known to play a role in the entry of fungi into host plant cells (proteases). Fourth, *O. maius* F3860 is able to secrete enzymes that release mineral elements from soil compounds (phytases and proteases), as well as auxins, which have been repeatedly noted as positive qualities for microorganisms that stimulate plant growth.

Thus, we found in *O. maius* F3860 a set of characteristics that are attractive from the standpoint of heather plant biotechnology. Further research will be aimed at elucidating the effects of *O. maius* F3860 when co-cultivated with heather plants.

## Figures and Tables

**Table 1:** Rates of enlargement of *O. maius* F3860 colonies’ diameters on different nutrient media.

Nutrient Media	K_D_	Reliability Parameter for Linear Approximation R²
CDA	0.4661	0.9741
And	0.7230	0.9900
WPM	0.6612	0.9942
MEA	0.9624	0.9942
PDA	1.0878	0.9943
CDA + EtOH	0.2638	0.9249
CDA + R	0.3445	0.9811
CDA + Q	0.3382	0.9704

CDA—Czapek-Dox agar, And—Anderson medium, WPM—Woody Plant medium, MEA—malt extract agar, PDA—potato dextrose agar, CDA + EtOH—Czapek-Dox agar with ethanol, CDA + R—Czapek-Dox agar with rutin ethanolic solution (final concentration of 10 μM), CDA + Q—Czapek-Dox agar with quercetin ethanolic solution (final concentration of 10 μM).

## Data Availability

Data are contained within the article and Supplementary Material.

## Supplementary Materials

The following supporting information can be downloaded at: https://www.mdpi.com/article/10.3390/jof9070728/s1. Dataset S1: ITS sequence of the *O. maius* F3860.
