# Supplementary material for: Several Characteristics of Oidiodendron maius G.L. Barron Important for Heather Plants’ Controlled Mycorrhization
Source: J Fungi (Basel). 2023 Jul 5;9(7):728. doi: 10.3390/jof9070728 (PMC10381259; doi:10.3390/jof9070728)
Supplement: Supplementary file 1 [file jof-09-00728-s001.zip › jof-2407468-supplementary.pdf]

Supplementary Dataset. ITS sequence of the *O. maius* F3860

CTGCGGAGGGATCATTACAGAGTTCATGCCCTCCGGGTAGATCTCCCACCCATTGCTATCACTAC
TCTCGTTGCTTTGGCGGGCCGCTGGGCCCTGCCCGGCCGCCGGCCCCGGCTGGCGCGCGCCCGCC
AGAGACCTCACAGACTCTGAATGTTAGTGTCGTCCGAGTAACTATATAATCGTTAAAACTTTCAA
CAACGGATCTCTTGGTTCTGGCATCGATGAAGAACGCAGCGAAATGCGATAAGTAATGCGAATT
GCAGAATTCAGTGAGTCATCGAATCTTTGAACGCACATTGCGCCCTGTGGTATTCCGCAGGGCAT
GCCTGTTCGAGCGTCATTTCAACCCTCAAGCCTCGCTTGGTGTTGGGCCCTGCCCGTCGCGGCCGG
CCCTAAAGATAGTGGCGGCGCCGCCTGGCCCTCAGCGTAGTACAGCTCTCGCTCCAGGGTCCGG
CGGCAGCCTGCCAGAACCCCCCAACTCTTGTGGTTGACCTCGGATCAGGTAGGGATACCCGCTG
AACTTAAGCATATC
